# Identification and Functional Annotation of Genes Related to Bone Stability in Laying Hens Using Random Forests

**DOI:** 10.3390/genes12050702

**Published:** 2021-05-08

**Authors:** Simon Jansen, Ulrich Baulain, Christin Habig, Faisal Ramzan, Jens Schauer, Armin Otto Schmitt, Armin Manfred Scholz, Ahmad Reza Sharifi, Annett Weigend, Steffen Weigend

**Affiliations:** 1 Institute of Farm Animal Genetics, Friedrich-Loeffler-Institut, 31535 Neustadt, Germany; ulrich.baulain@fli.de (U.B.); christin.habig@fli.de (C.H.); jens.schauer@fli.de (J.S.); annett.weigend@fli.de (A.W.); steffen.weigend@fli.de (S.W.); 2 Breeding Informatics Group, Department of Animal Sciences, University of Göttingen, 37075 Göttingen, Germany; faisal.ramzan@stud.uni-goettingen.de (F.R.); armin.schmitt@uni-goettingen.de (A.O.S.); 3 Center for Integrated Breeding Research (CiBreed), University of Göttingen, 37075 Göttingen, Germany; rsharif@uni-goettingen.de; 4 Livestock Center of the Faculty of Veterinary Medicine, Ludwig-Maximilians-University Munich, 85764 Oberschleissheim, Germany; armin.scholz@lvg.vetmed.uni-muenchen.de; 5 Animal Breeding and Genetics Group, Department of Animal Sciences, University of Göttingen, 37075 Göttingen, Germany

**Keywords:** bone mineral density, bone breaking strength, gene set enrichment analysis, osteoporosis, Random Forests, single nucleotide polymorphism, skeletal integrity

## Abstract

Skeletal disorders, including fractures and osteoporosis, in laying hens cause major welfare and economic problems. Although genetics have been shown to play a key role in bone integrity, little is yet known about the underlying genetic architecture of the traits. This study aimed to identify genes associated with bone breaking strength and bone mineral density of the tibiotarsus and the humerus in laying hens. Potentially informative single nucleotide polymorphisms (SNP) were identified using Random Forests classification. We then searched for genes known to be related to bone stability in close proximity to the SNPs and identified 16 potential candidates. Some of them had human orthologues. Based on our findings, we can support the assumption that multiple genes determine bone strength, with each of them having a rather small effect, as illustrated by our SNP effect estimates. Furthermore, the enrichment analysis showed that some of these candidates are involved in metabolic pathways critical for bone integrity. In conclusion, the identified candidates represent genes that may play a role in the bone integrity of chickens. Although further studies are needed to determine causality, the genes reported here are promising in terms of alleviating bone disorders in laying hens.

## 1. Introduction

The very high incidence of skeletal disorders in laying hens, including brittle and fractured bones, is undoubtedly one of the most serious problems facing the egg production industry [1,2]. Bone demineralisation associated with eggshell calcification favours the loss of structural bone tissue and ultimately predisposes the birds to osteoporosis in the course of the laying period [3,4]. Besides dramatic effects on animal welfare [5,6,7], bone weakness also has an economic impact [4,8]. According to a widespread assumption, the reduction in bone stability is primarily the result of selection for high laying performance [9,10,11]. However, the role of genetic selection on egg production is now seen in a more differentiated view, with recent studies pointing to factors other than egg number alone [12,13,14].

In the urgently needed improvement of the skeletal health of laying hens, genetics play an important role alongside husbandry and feeding of the birds [3,15,16]. To date, a number of quantitative trait loci (QTL) have been mapped to skeletal traits in chickens [17,18,19,20,21,22]. Dunn et al. [23] discovered a QTL on chromosome 1 that was recently fine-mapped leading to the identification of a promising region around the *cystathionine beta synthase* gene associated with osteoporosis [24]. The discovery of candidate positions for bone integrity is inevitably linked to technical advances in genotyping and bioinformatics. Today, testing hundreds of thousands of single nucleotide polymorphisms (SNP) by means of genome-wide association studies (GWAS) has become common practice [15,25,26].

Despite its widespread use, GWAS has some potential pitfalls. In addition to population stratification, these include the identification of gene loci with small effect sizes, which rarely reach the statistical significance level due to their low strength of association [27]. At this point, machine learning algorithms represent a promising advance. Several studies have demonstrated their potential in identifying genes with small effect sizes [28,29]. The Random Forests (RF) models in particular seem to have a great potential for analysing a large number of loci simultaneously and identifying corresponding associations [29,30,31]. Recently, this approach has been used to identify genes associated with eggshell strength [27].

The aim of the current study was to identify genomic positions associated with bone stability traits, i.e., breaking strength and mineral density of the tibiotarsus and the humerus, in laying hens. The animal model used comprised four layer lines that differed in their phylogenetic origin (brown-egg vs. white-egg layers) and their egg production level (high vs. moderately performing lines) [32]. Jansen et al. [14] have recently reported promising heritability estimates for bone traits in this set of populations, supporting the assumption of an inherited component of hens’ susceptibility to osteoporosis. In the study reported here, we took a deeper look into the underlying genetic architecture of these hens. This includes the adoption of RF-based feature selection in order to find potentially important SNPs. Subsequently, we performed a series of functional analyses including gene set enrichment analysis. Furthermore, SNP effects were estimated to confirm candidate genes known from the literature to be associated with bone metabolism.

## 2. Materials and Methods

### 2.1. Population and Experimental Setup

The population consisted of four purebred chicken layer lines (*Gallus gallus domesticus*), which are phylogenetically distinct (brown- vs. white-egg lines). Within each of these phylogenetic groups, the two lines differed in terms of egg-laying rate (high- vs. moderate-performing lines) [32,33]. The set of populations was previously subjected to phenotypic analysis and the estimation of genetic parameters [14]. The data set only comprised hens whose total egg number was within the line specific threefold interquartile range and who laid at least one egg from 67 to 69 weeks of age [14]. For the statistical analyses done in this study, we combined the four chicken lines into one set as described below.

For the current research, we used the bone breaking strength (BBS) and bone mineral density (BMD) measurements previously reported by Jansen et al. [14]. A summary of these measurements, taken from reference [14], is presented in Table S1. Briefly, BBS and BMD of the tibiotarsus and humerus were determined by the three-point bending test and dual-energy X-ray absorptiometry, respectively, using dissected bones after the hens were sacrificed at 69 weeks of age.

**Figure 1:**
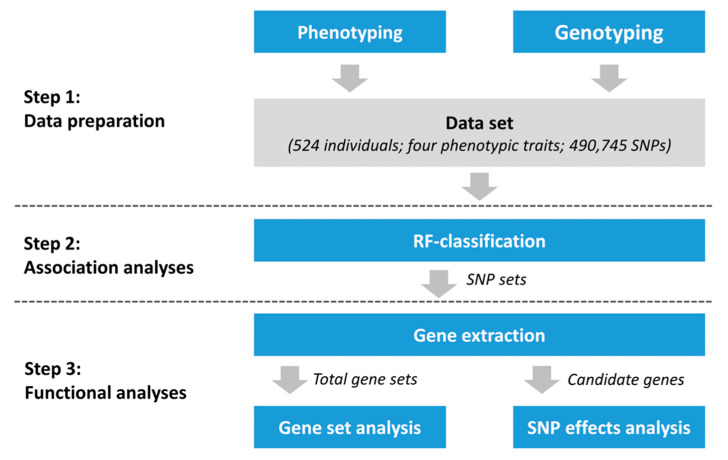
Schematic illustration of the study design. The data set included 524 laying hens phenotyped for bone stability traits. The corresponding genotypes included 490,745 SNP markers. Association analysis was performed applying Random Forests (RF) classification. Genes harbouring significant SNPs were extracted and screened for links to bone stability. Gene set analyses were performed considering all genes obtained from the RF classification. Retrospectively, SNP effects were estimated for a subset of candidate genes identified in gene sets obtained from the RF classifier.

The experimental setup is shown in Figure 1. We applied the machine learning-based approach of Random Forests to identify genomic positions potentially associated with the given phenotypes. Subsequent functional analyses included gene set enrichment analysis and retrospective SNP effects analysis.

### 2.2. Genotyping

Initially, deoxyribonucleic acid (DNA) samples from the hens and sires were extracted from blood samples. The hens were genotyped for 51,837 SNPs with a custom-made SNP array (Affymetrix Inc., Santa Clara, CA, USA). From the same chicken lines, in total 80 sires were genotyped for 580,961 SNP markers using the Affymetrix^®^ Axiom^®^ Genome-Wide Chicken Genotyping Array [34]. Quality control was applied to both data sets using the SNP & Variation Suite (SVS) v8.9 [35]. We only considered SNPs from autosomal chromosomes 1 to 28. The genotypes were filtered for a SNP call rate of ≥99% and an animal call rate of ≥95%. Furthermore, missing genotypes were imputed in a two-step procedure using BEAGLE 5.0 [36]. Missing markers within the sire data set were imputed using the default settings. After this, the female genotypes were imputed from 37,606 SNPs left after quality control to 497,041 SNPs. Here, the sire genotypes served as a reference population and the effective population size was set to *ne* = 5000. After imputation, 524 hens and 497,041 SNPs remained, of which 490,745 SNPs were finally annotated using the genome assembly GRCg6a (galGal6) [37], with duplicated SNPs and those with ambiguous chromosome annotation being removed.

### 2.3. Random Forests Classification

We applied the machine learning algorithm of Random Forests (RF) to identify SNPs associated with bone characteristics, i.e., BBS and BMD of the tibiotarsus and humerus. Briefly, the RF algorithm constructs a multitude of classifying decision trees assigning importance values to each SNP, thus determining those SNPs that explain variation in the response variable [29]. As shown by Ramzan et al. [27,38], we performed SNP selection by applying the Boruta algorithm, which works as a wrapper around the classification algorithm [39]. This algorithm is based on the idea that an unimportant attribute is not more useful for classification than a random one. Hence, if an attribute shows lower importance than a random attribute, it can be deemed irrelevant. The second idea is that importance measures get more accurate with less irrelevant attributes, such that iteratively removing unimportant attributes increases the accuracy of the importance measure. The procedure of the algorithm is as follows: The dataset is first expanded by adding shuffled copies of the original values of each SNP, called shadow attributes. RF classification is then applied iteratively, assigning a value to each SNP, which is considered as the importance of the SNP. At each iteration, SNPs whose importance is less than the best of their shadow attributes are removed.

We used the Python (v3.8.3) [40] implementation from Homola [41] that specifies the proportion of the shadow attributes by which a SNP has to be better in order to be selected as important. Embedded in the Boruta algorithm, the RF classification itself was carried out with the ‘RandomForestRegressor’ from the Scikit-learn package [42] using default settings. The parameter *perc* was set to 99, representing a threshold of 99%, as no SNP has been confirmed as important at the 100% level. RF classification was performed separately for each bone trait. The input file consisted of the SNP genotypes, coded as ‘0’ (AA), ‘1’ (AB), or ‘2’ (BB), and the phenotypic values of the respective bone trait. To account for possible confounding effects due to population stratification, residuals representing adjusted phenotypes were analysed instead of the raw values [29]. The following model was used to estimate the residuals:

(1) $$\gamma_{ijkl}=\mu +G_{i}+LL_{j}+S_{k}+\varepsilon_{ijkl}$$

where $\gamma_{ijkl}$ is the observation for a bone trait, μ is the general mean, $G_{i}$ is the fixed effect of generation (i = 1, 2), $LL_{j}$ is the fixed effect of layer line (j = 1 to 4), $S_{k}$ is the random effect of sire (k = 1 to 145), and $\varepsilon_{ijkl}$ is the residual error. The model was computed using JMP v14.0 (SAS Institute Inc. Cary, NC, USA, 2018). Normal distribution of the residuals was assumed (Figure S1).

The output of the RF classification was a list of confirmed SNPs, i.e., markers that are more than coincidentally associated with a given bone trait.

### 2.4. Functional Analyses

#### 2.4.1. Gene Extraction

All steps of the functional analyses were carried out using R v4.0.3 [43]. Extraction of genes associated with SNPs identified by the RF classification from the Ensembl database v102 [44] was performed using BioMart [45]. All protein-coding genes that are located within 5 kb upstream and downstream of the respective SNPs were considered for the gene lists. Information on the biological functions of these genes was obtained from both the NCBI [46] and Ensembl databases, as well as from the literature. The gene lists were then screened for genes known to be associated with bone stability traits. In this way, we identified a number of genes that were henceforth regarded as candidate genes.

#### 2.4.2. SNP Effects Analysis

The genotypic effect was analysed for those SNPs located in intragenic or in flanking genomic regions of candidate genes, which have previously been shown to be significantly associated with a bone trait (see Table 1). SNP effects for each locus were analysed as described by Wiedemann et al. [47]. For this purpose, the actual SNP genotypes were coded as ‘0’ (AA), ‘1’ (AB), or ‘2’ (BB), with the B allele representing the minor allele. The minor allele was considered the effect allele, whereas the major allele was termed ‘other allele’. All models were computed with the R package lme4 [48].

A linear regression model adjusted for fixed factors was applied to estimate the allele substitution effects by single marker regression (SMR):

(2) $$\gamma_{ijklm}=\mu +G_{i}+LL_{j}+b_{1}SNP_{k}+S_{l}+\varepsilon_{ijklm}$$

where $\gamma_{ijklm}$ is the observation for a bone trait, μ is the overall mean effect, $G_{i}$ is the fixed effect of generation (i = 1, 2), $LL_{j}$ is the fixed effect of layer line (j = 1 to 4),$\;b_{1}$ is the regression coefficient of the SNP genotype ($SNP_{k}$), $S_{l}$ is the random effect of sire (l = 1 to 145), and $\varepsilon_{ijklm}$ is the residual error. Standardised allele substitution effects were calculated according to model (2) after both the dependent variable and the SNP genotypes coded as ‘0’, ‘1’, or ‘2’ were standardised to have a mean of 0 and a standard deviation of 1.

To calculate the additive and dominance effects, a dominant-recessive model (DRM) was applied considering the SNP genotype as a fixed class variable. The statistical model was as follows:

(3) $$\gamma_{ijklm}=\mu +G_{i}+LL_{j}+SNP_{k}+S_{l}+\varepsilon_{ijklm}$$

where $\gamma_{ijklm}$ is the observation for a bone trait, μ is the overall mean effect, $G_{i}$ is the fixed effect of generation (i = 1, 2), $LL_{j}$ is the fixed effect of layer line (j = 1 to 4), $SNP_{k}$ is the fixed effect of SNP genotype (k = 1 to 3), $S_{l}$ is the random effect of sire (l = 1 to 145), and $\varepsilon_{ijklm}$ is the residual error. Least squares means (LSM) for the different genotypes were estimated with the emmeans package [49]. Significant differences between LSM were tested using a *t*-test and adjusted by the Bonferroni method. Additive and dominance effects were estimated by contrasting the respective genotypes according to the following formulas.

(4) $$a=\frac{\mu_{AA}-\mu_{BB}}{2}$$

(5) $$d=\mu_{AB}-\frac{\mu_{AA}+\mu_{BB}}{2}$$

where a is the additive effect, d is the dominance effect, $\mu_{AA}$ and $\mu_{BB}$ are the phenotypic mean values of the homozygous genotypes, and $\mu_{AB}$ is the phenotypic mean value of the heterozygous genotype.

#### 2.4.3. Gene Set Analysis

With the gene sets including all genes extracted, we performed gene set analysis (GSA) using g:Profiler2 [50]. This involved the Gene Ontology (GO) (Ensembl v102) and the Kyoto Encyclopedia of Genes and Genomes (KEGG) [51] (FTP release 2020-09-07) databases. The GSA was carried out considering all known genes obtained from Ensembl for the calculation of statistical significance and applying the default g: SCS algorithm [52] for computing the multiple testing correction. Only GO- and pathway terms with significant enrichment (*p* < 0.05) were considered for further analyses. Tree maps of the GO terms were generated using rrvgo [53].

**Table 1:** General information for 17 loci associated with the bone breaking strengths (BBS) or bone mineral densities (BMD) of the tibiotarsus (Tib) and humerus (Hum) selected for the SNP effects analysis.

SNP	Trait	Location	GGA ^1^	Position ^2^	Genotypes	N Individuals	Genotype Frequencies	EA/OA ^3^	EA Frequency	Candidate Gene	Reference ^4^
AX-75268181	Tib_BMD	intragenic	1	139,001,157	CC/CT/TT	392/96/36	0.75/0.18/0.07	T/C	0.16	*MCF2L*	[54]
AX-76044166	Tib_BBS	intragenic	2	15,440,861	AA/AG/GG	421/63/40	0.80/0.12/0.08	G/A	0.14	*MPP7*	[55]
AX-80813610	Tib_BMD	downstream	2	23,056,581	CC/CG/GG	339/113/72	0.65/0.22/0.13	G/C	0.25	*CALCR*	[56]
AX-76099065	Tib_BMD	intragenic	2	46,101,680	GG/GA/AA	392/77/55	0.75/0.15/0.10	A/G	0.18	*SFRP4*	[57]
AX-76601713	Tib_BBS	intragenic	3	10,617,925	AA/AG/GG	265/102/157	0.51/0.19/0.30	G/A	0.40	*ACTR2*	[15]
AX-77276717	Tib_BBS	intragenic	3	19,498,104	GG/GA/AA	322/145/57	0.61/0.28/0.11	A/G	0.25	*TGFB2*	[58]
AX-76491534	Tib_BBS	intragenic	3	49,027,160	AA/AG/GG	432/62/30	0.82/0.12/0.06	G/A	0.12	*CCDC170*	[59]
AX-76772658	Tib_BBS/Hum_BBS	intragenic	5	11,438,677	TT/TC/CC	219/199/109	0.41/0.38/0.21	C/T	0.40	*SOX6*	[60]
AX-77113061	Tib_BMD	upstream	8	5,889,886	GG/AG/AA	202/156/166	0.38/0.30/0.32	A/G	0.47	*TMCO1*	[61]
AX-77091655	Hum_BBS/Hum_BMD	upstream	8	24,931,025	CC/CA/AA	286/139/99	0.54/0.27/0.19	A/C	0.32	*PODN*	[15]
AX-75597497	Hum_BBS	downstream	10	19,108,829	AA/AG/GG	376/124/24	0.72/0.24/0.04	G/A	0.16	*SMAD6*	[62]
AX-75677174	Tib_BMD	intragenic	11	10,044,055	CC/CT/TT	377/107/40	0.72/020/0.08	T/C	0.18	*GPATCH1*	[55]
AX-75711229	Tib_BBS	intragenic	12	3,804,145	GG/AG/AA	459/58/7	0.88/0.11/0.01	A/G	0.07	*ASPN*	[63]
AX-75913642	Tib_BBS	upstream	18	8,793,585	GG/AG/AA	451/61/12	0.86/0.12/0.02	A/G	0.08	*SOX9*	[64]
AX-76351785	Hum_BBS	intragenic	27	3,497,444	CC/CT/TT	316/138/70	0.61/0.26/0.13	T/C	0.26	*WNT9B*	[65]
AX-76351898	Hum_BMD	downstream	27	3,518,924	GG/GA/AA	483/31/10	0.92/0.06/0.02	A/G	0.05	*WNT3*	[55]
AX-76351899	Hum_BMD	downstream	27	3,519,091	TT/TC/CC	483/31/10	0.92/0.06/0.02	C/T	0.05	*WNT3*	[55]

^1^ GGA, Gallus gallus chromosome; ^2^ Physical position (bp) according to the GRCg6a (galGal6) genome assembly; ^3^ EA, effect allele (minor allele); OA, other allele (major allele); ^4^ References from the literature suggesting an association of the gene with bone stability traits.

## 3. Results

### 3.1. Identified Single Nucleotide Polymorphisms

Lists of confirmed SNPs were obtained from the RF classifier for each of the phenotypic traits. For the tibiotarsus, 358 (BBS) and 374 (BMD) SNPs were confirmed as important, whereas for the humerus 188 (BBS) and 178 (BMD) markers were identified, respectively. There were no confirmed SNPs on GGA (*Gallus gallus* chromosome) 16 for any of the four traits studied (Figure S2). In the case of the tibiotarsus, the majority of SNPs were located on GGA 1. In general, there were fewer markers for the humerus, with no markers found on GGA 28. Comparing the two bone types, more than twice as many SNPs were identified for the tibiotarsus.

### 3.2. Functional Analyses

#### 3.2.1. Extracted Gene Sets

We identified 240 (BBS) and 220 (BMD) genes within an interval of 5 kb upstream and downstream of SNPs that were found to be significant for the tibiotarsus. In contrast, gene sets for the humerus included 115 (BBS) and 113 (BMD) genes.

**Figure 2:**
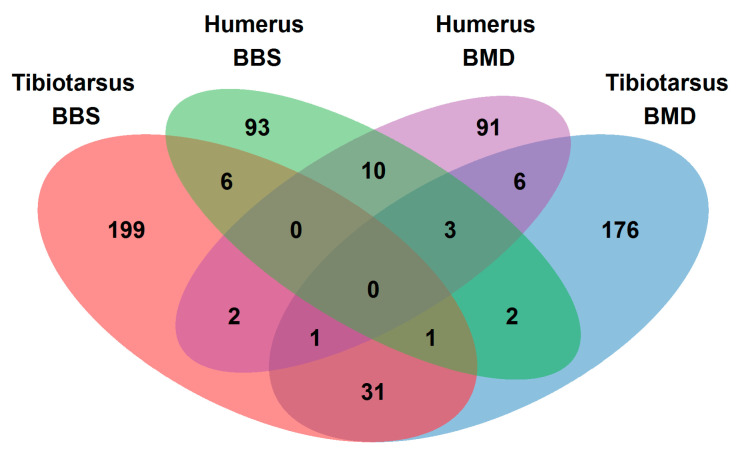
Venn diagram showing the overlap of genes for the bone breaking strengths (BBS) and bone mineral densities (BMD) of the tibiotarsus and humerus.

A Venn diagram was drawn to find overlaps and differences between the genes identified for the BBS and BMD of the two bone types (Figure 2). The corresponding detailed gene list is given in Table S2. It was found that the overlaps of loci between the individual traits were rather small. It ranged from 1.7% (six genes) between BBS of tibiotarsus and humerus up to an overlap of 6.7% (31 genes) between BBS and BMD within the tibiotarsus. No gene was found in all bone and trait combinations. Rather, they were mainly unique genes.

Based on the information on their biological functions and from the literature review, we reduced the gene lists to genes that are known to be related to bone stability. We found 16 genes with an already described association (Table 1). These genes are located on GGA 1 (*MCF2L*), GGA 2 (*MPP7, CALCR, and SFRP4*), GGA 3 (*ACTR2*, *TGFB2*, and *CCDC170*), GGA 5 (*SOX6*), GGA 8 (*TMCO1*, *PODN*), GGA 10 (*SMAD6*), GGA 11 (*GPATCH1*), GGA 12 (*ASPN*), GGA 18 (*SOX9*), and GGA 27 (*WNT9B*, *WNT3*).

#### 3.2.2. SNP Effects Analysis

To reveal the biological significance of the candidate genes, we analysed their associations with the corresponding phenotypic bone traits. To this end, we performed SNP effects analyses of all markers detected by the RF classifier and then assigned to genes (Table 1). Since the SNPs *AX-77091655* (*PODN*) and *AX-76772658* (*SOX6*) were associated with two traits each and, in addition, two further markers were assigned to the *WNT3* gene, SNP effects were estimated for 19 SNP and bone trait combinations.

Results from the SMR model are shown in Table 2. Analysis of variance revealed significant effects of SNP genotypes on the respective bone traits. Only the SNPs *AX-77276717* (*TGFB2*) and *AX-75711229* (*ASPN*) had no significant effect. Locus *AX-76099065* (*SFRP4*) had the greatest effect on tibiotarsus BMD, with the substitution of allele G for allele A leading to a reduction of 0.016 g/cm^2^. In contrast, increasing the number of the copies of the effect alleles at loci *AX-76351898* and *AX-76351899*, both assigned to the *WNT3* gene, would yield an increase in humerus BMD of 0.016 g/cm^2^. Of all loci significantly associated with BBS, the SNP *AX-76491534* (*CCDC170*) showed the largest effect, whereby substitution of one copy of allele A with allele G would result in a 15.63 N decrease of tibiotarsus BBS. The counterpart is the SNP *AX-76351785* (*WNT9B*) in which the T allele would presumably cause an increase of 11.51 N of humerus BBS. This is also the largest effect among all significant SNPs, with a change of 0.21 expressed in SD units.

Results obtained from the DRM are shown in Table 3. Comparison of the genotypic values (LSM) revealed significant differences among the genotypes. This applies to all loci studied, with exception of the SNPs *AX-77276717* (*TGFB2*) and *AX-80813610* (*CALCR*), where only a tendency towards a higher value for the homozygote genotype of the effect allele was observed. For the other loci, the effects indicated by the direction of the beta coefficients (SMR) were also reflected in the genotypic values. A significantly higher LSM was found for the homozygote genotype of the effect allele of the SNP *AX-75711229* (*ASPN*). However, this estimate might be biased as the corresponding genotype had a frequency of only 0.01 (Table 1) and no significant allele substitution effect was detected for this locus (Table 2).

Significant additive effects of the respective other allele (major allele) were accounted for all loci with exception of the SNPs *AX-77276717* (*TGFB2*) and *AX-76772658* (*SOX6*) (Table 3). The estimates ranged from −0.02 to 0.017 g/cm^2^ for the BMD-related SNPs and from −16.70 to 15.70 N for the markers associated with the BBS. Effects of complete dominance were observed for the SNPs *AX-76044166* (*MPP7*), *AX-75711229* (*ASPN*), *AX-75597497* (*SMAD6*) and *AX-76099065* (*SFRP4*), with one copy of the major allele masking the recessive allele, thus leading to full trait expression. In contrast, complete dominance in favour of the effect allele was seen for the SNP *AX-77113061* (*TMCO1*).

#### 3.2.3. Gene Set Analysis

GSA was performed considering the total gene sets. We restricted the results presented to the GO biological process (BP) category, as we sought to determine overarching biological objectives to which the gene products of the extracted genes contribute. Furthermore, the genes were grouped according to their KEGG pathways. Full lists of significantly enriched GO terms, including those from the cellular component and molecular function categories, are given in Table S3.

**Figure 3:**
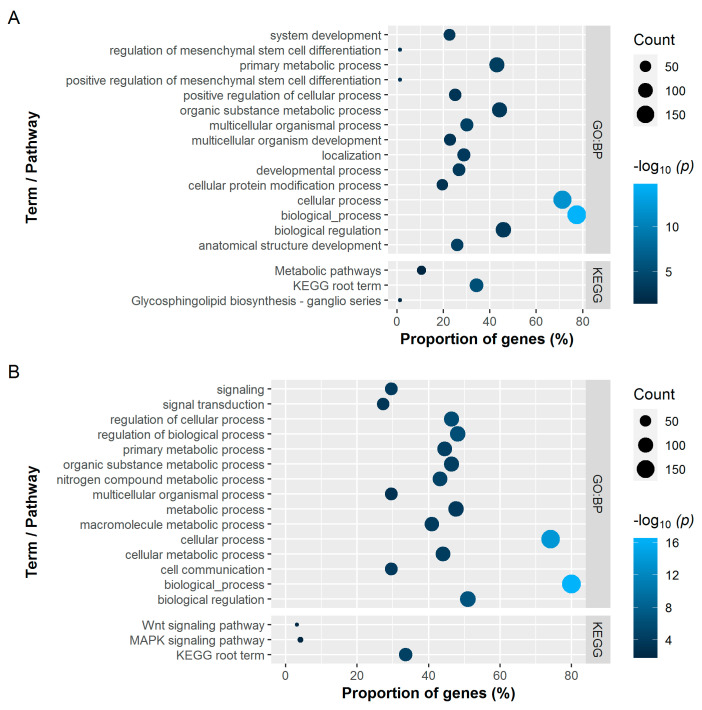
Significantly enriched Gene Ontology terms of the category biological processes (GO:BP; top 15 with the highest -log_10_ *p*-values) and KEGG pathways for the bone breaking strength (**A**) and bone mineral density (**B**) of the tibiotarsus. The dot size represents the absolute number of genes enriched in the term. The proportion of enriched genes in all queried genes is represented on the *x*-axis. The colour represents the −log_10_ transformed adjusted *p*-values.

**Figure 4:**
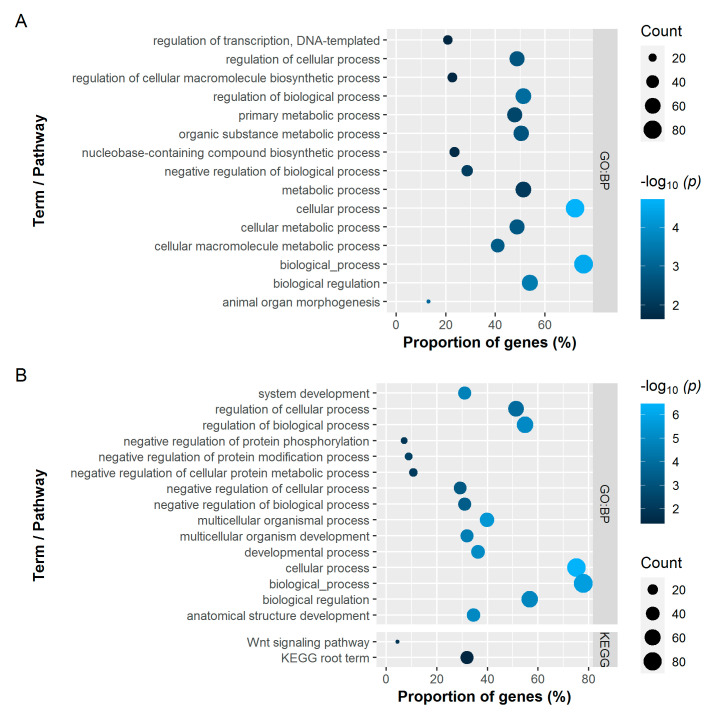
Significantly enriched Gene Ontology terms of the category biological processes (GO:BP; top 15 with the highest -log_10_ *p*-values) and KEGG pathways for the bone breaking strength (**A**) and bone mineral density (**B**) of the humerus. The dot size represents the absolute number of genes enriched in the term. The proportion of enriched genes in all queried genes is represented on the *x*-axis. The colour represents the −log_10_ transformed adjusted *p*-values.

A large number of genes were involved in common processes. The analysis reported 81 (BBS) and 51 (BMD) significantly enriched BPs for the tibiotarsus and 33 (BBS) and 42 (BMD) BPs for the humerus, respectively (Table S3). Of these, Figure 3 (tibiotarsus) and Figure 4 (humerus) show the top 15 significantly enriched GO BP terms with the highest −log_10_ *p*-values and all significantly enriched KEGG pathways obtained from the RF classifier. Although certain BPs overlapped between the bone and trait combinations, no relation to the skeletal system was evident in the enriched BPs. Visualizing the results using tree maps to investigate redundancy based on semantic similarity between different GO terms also did not yield any biologically relevant findings (Figures S3 and S4). However, the literature points to the involvement of Wnt- and MAPK signalling pathways in the pathogenesis of osteoporosis [25]. GSA revealed the *Wnt signaling pathway* (KEGG:04310) to be significantly enriched in both BMD gene sets (Figure 3B and Figure 4B). In addition, significant enrichment for the *MAPK signaling pathway* (KEGG:04010) was identified in the genes for BMD of the tibiotarsus.

## 4. Discussion

The objective of the present study was to identify genomic positions potentially associated with skeletal integrity in a laying hen population. There is solid evidence that osteoporosis is a polygenic disorder, i.e., determined by multiple functional genes acting conjointly rather than a few major genes [15,25]. For this reason, we applied RF classification, an approach known to be able to detect genes with modest effects [29,30]. To our knowledge, this is the first study applying a machine-learning approach to bone data in chickens. Using RF classification, we identified a high number of potentially informative SNPs. Although a large number of genes were adjacent to these SNPs, only 16 candidate genes related to skeletal disorders were identified; of these, many had human orthologues. However, for the vast majority of genes, no involvement in bone metabolism has been suspected so far, which is in line with previous reports [15,66]. From the 16 identified candidates discussed below, we first focus on genes that have previously been linked to BBS or BMD (*n* = 10), followed by genes for which an association with osteoarthritis is suggested (*n* = 3). Finally, genes are discussed that are functionally related to the Wnt signalling pathway (*n* = 3).

Ten of our candidate genes can be grouped as having previously been associated with BBS or BMD traits in the literature. Of these, the *membrane palmitoylated protein 7* gene (*MPP7*) was associated with vertebral BMD in humans [55]. Its strong functional role in osteoblast biology was demonstrated by means of in vivo and in vitro studies [67]. Based on these reports, we consider *MPP7* to be a good candidate for bone disorders in chickens. In our study, the *calcitonin receptor gene* (*CALCR*) was identified as a strong candidate for BMD. Calcitonin plays a role in calcium homeostasis and is primarily an inhibitor of bone resorption [68]. Our observations are in line with previous reports, as *CALCR* polymorphisms were associated with site-specific BMD in humans [56,69], and alpha-calcitonin gene-related peptide deficient mice were shown to have a lower bone mass [70]. One of the candidates for BBS located on GGA 3 is the *actin related protein 2* gene (*ACTR2*), which was recently identified by Raymond et al. [15] as being associated with BBS in laying hens. *ACTR2* is functionally linked to bone via its importance for cilia formation, as cilia are known to play an integral role in skeletal development [15,71]. Although no significant effect of the variant corresponding to the *transforming growth factor beta 2* gene (*TGFB2*) was observed in our study, *TGFB2* is considered a very promising candidate for skeletal integrity in the chicken. As a cytokine, the protein encoded by *TGFB2* has important functions in many biological processes related to bone remodelling [19,58]. Analyses in different chicken populations including broilers and layers suggest *TGFB2* to be associated with various bone characteristics [19,21,58]. In this context, the *SMAD family member 6* gene (*SMAD6*) has to be mentioned, which we identified as a candidate for BBS. Its protein acts as a regulator of the TGF-beta family and inhibits bone morphogenetic protein pathways, which are integral parts of osteoblast and chondrocyte differentiation [72,73]. A study on mice revealed their essential role in bone formation, as *SMAD3* knockout resulted in osteopenia [62]. The *coiled-coil domain containing 170* gene (*CCDC170*) is our third candidate for BBS located on GGA 3. The region around this locus has been linked to BMD in humans [59,65]. However, since the function of the protein is yet unclear, it has been speculated whether associations attributed to *CCDC170* do not rather belong to the adjacent *estrogen receptor 1* gene [59]. In a follow-up study, *CCDC170* polymorphisms were in turn associated with osteoporosis-relevant phenotypes [74]. Only one of our candidates was located on GGA 5. The corresponding variant is located in the intron of the *SRY-box 6* gene (*SOX6*), which encodes a transcription factor known to affect developmental processes and skeletal formation in humans [60,65]. In addition, the gene was linked with BMD of the femoral neck [75], and skeletal abnormalities have previously been observed in *SOX6* knockout mice, suggesting an integral role in cartilage formation [76]. We identified the *transmembrane and coiled-coil domains 1* gene (*TMCO1*), located on GGA 8, as a candidate for BMD. *TMCO1* plays an important role in bone formation-mediating calcium homeostasis within the endoplasmic reticulum [61]. Disruption of the endoplasmic reticulum of an osteoblast can lead to severe bone disorders [77]. Recently, Li et al. [61] demonstrated that *TMCO1* deficiency leads to reduced bone formation and osteoblast differentiation in humans and mice. In addition to *SOX6*, the *podocan* gene (*PODN*) is another candidate that was associated with two traits, namely BBS and BMD of the humerus. *PODN* encodes a proteoglycan that was shown to bind type 1 collagen, suggesting a potential role in growth regulation [78]. At this point, the great influence of collagen on mechanical properties of bones should be mentioned, which is assumed to apply equally to humans [79] and chickens [80]. That *PODN* could be a promising candidate for bone integrity in laying hens is supported by findings of Raymond et al. [15]. Although the *G-patch domain containing 1 gene* (*GPATCH1*), identified as BMD candidate, is considered a candidate gene for osteoporosis in humans [55], functional information is limited and its role in skeletal pathophysiology is not yet clear.

For a group of three candidates, the literature suggests a functional relationship with osteoarthritis, a pathological condition of cartilage degradation [81]. Osteoarthritis and osteoporosis are closely related and characterised by subchondral bone loss and excessive bone resorption [20,81,82]. It is assumed that both diseases are partly determined by common genes [83]. One of the candidates found in our study is the *MCF.2 cell line derived transforming sequence like* gene (*MCF2L*), shown to be expressed in cartilage tissue, and linked to joint osteoarthritis in humans [54,84]. In addition, Mao et al. [85] recently pointed out the relevance of *MCF2L* for osteoporosis, which underlines the link between both disorders. The *asporin* gene (*ASPN*), also known as *biglycan* (*BGN*), is assumed to regulate chondrogenesis. While the results of Mishra et al. [63] point to a functional role of *ASPN* in osteoarthritis, other studies reported only a marginal relationship or contradict such an association [86,87]. Given these contradictory results and the fact that the association with *ASPN* was not significant in our study, we consider *ASPN* a suggestive candidate that requires further investigation. The *SRY-box 9* gene (*SOX9*) is our third candidate linked to osteoarthritis [88]. *SOX9* is considered a pivotal player in chondrogenesis, as its protein, the transcription factor SOX-9, was shown to stimulate chondrocyte differentiation [64,89]. In addition, *SOX9* mediates the Wnt signalling pathway, abnormalities of which are correlated with cartilage degradation [64].

The remaining candidates, i.e., the *SFRP4*, *WNT3*, and *WNT9B* genes, are functionally linked to the Wnt signalling pathway, which plays a key role in various basic developmental processes [90]. The *secreted frizzled related protein 4* gene (*SFRP4*) encodes a protein that primarily antagonizes Wnt polypeptides [90] and is one of the BMD candidates. A mutation in *SFRP4* was shown to cause pathological reduction of cortical bone tissue in mice and humans [57]. The Wnt signalling pathway is crucial for bone metabolism and to date, several Wnt genes are known to be associated with traits such as bone mass and BMD [55,91]. This also includes the *Wnt family member 3* gene (*WNT3*), which was identified in this study [55,88]. The *Wnt family member 9B* gene (*WNT9B*), located adjacent to *WNT3* on GGA 27, was identified as a candidate for BBS. Although its role in skeletal biology is less explored than that of other Wnt genes, we consider *WNT9B* a susceptibility gene for bone strength due to its association with femur BMD [65]. The high importance of the Wnt signalling pathway for bone strength is supported by the significant enrichment that was shown in the GSA for this functional pathway. Furthermore, the *mitogen-activated protein kinase (MAPK)* signalling pathway was enriched, which is also very important for skeletal development and, in particular, for chondrogenesis [92]. These observations are in accordance with recent results from pathway analyses [25,93].

Taken together, we identified a number of genetic loci associated with the bone traits studied. Based on these findings, we can confirm the assumption that bone stability is determined by multiple genes, each of which has a rather small effect size. The genes presented here represent suggestive susceptibility genes of bone integrity in chickens, some of which are nonetheless very promising based on what is known so far. Follow-up studies will be required to determine causalities and further uncover the biological significance of these genes. Here, the use of an F2 mapping population for high-resolution mapping of loci is recommended [94]. Considering the animal model, a follow-up study should also investigate the influence of phylogenetic origin on bone phenotypic plasticity, which was not done here, as we focused on finding loci that are significant for laying hens across phylogenetically divergent layer lines.

## 5. Conclusions

In this study, RF classification was performed to identify loci related with bone integrity in laying hens. In the subsequent functional analyses, a set of 16 promising candidate genes was identified, although in some cases rather small SNP effect estimates were observed. Some of the genes were shown to be involved in pivotal pathways that regulate bone metabolism. Our results strongly support genetics as a crucial factor that contributes significantly to the regulation of bone strength and thus offers great opportunities to improve bone health in laying hens. Further functional analyses on the candidate genes identified at a suggestive level have to follow in order to confirm their biological significance.

## Figures and Tables

**Table 2:** SNP effects analysis—Part 1: Analysis of variance table and allele substitution effect obtained from the single marker regression model.

SNP	Trait ^1^	Candidate Gene	Generation		Layer Line		SNP Genotype		Allele Substitution Effect ^2^			
			F-Statistics	*p*-Value	F-Statistics	*p*-Value	F-Statistics	*p*-Value	Beta (SE ^3^)	Standardised Beta ^4^ (SE)	*t*-Value	*p*-Value
AX-76044166	Tib_BBS	*MPP7*	80.92	<0.0001	46.34	<0.0001	4.05	0.0448	8.22 (4.09)	0.10 (0.05)	2.01	0.0448
AX-76601713	Tib_BBS	*ACTR2*	86.02	<0.0001	106.86	<0.0001	13.33	0.0003	−10.19 (2.79)	−0.18 (0.05)	−3.65	0.0003
AX-77276717	Tib_BBS	*TGFB2*	81.07	<0.0001	102.16	<0.0001	3.32	0.0696	4.67 (2.57)	0.06 (0.04)	1.82	0.0696
AX-76491534	Tib_BBS	*CCDC170*	91.49	<0.0001	84.86	<0.0001	12.58	0.0004	−15.63 (4.41)	−0.17 (0.05)	−3.55	0.0004
AX-76772658	Tib_BBS	*SOX6*	81.50	<0.0001	117.84	<0.0001	10.71	0.0012	7.63 (2.33)	0.12 (0.04)	3.27	0.0012
AX-75711229	Tib_BBS	*ASPN*	79.24	<0.0001	84.23	<0.0001	2.08	0.1503	6.66 (4.62)	0.05 (0.04)	1.44	0.1503
AX-75913642	Tib_BBS	*SOX9*	83.08	<0.0001	111.94	<0.0001	9.67	0.0019	−12.87 (4.14)	−0.11 (0.04)	−3.11	0.0019
AX-76772658	Hum_BBS	*SOX6*	36.26	<0.0001	52.59	<0.0001	5.67	0.0177	−5.32 (2.23)	−0.10 (0.04)	−2.38	0.0177
AX-77091655	Hum_BBS	*PODN*	39.91	<0.0001	41.64	<0.0001	8.35	0.0041	6.69 (2.31)	0.13 (0.04)	2.89	0.0041
AX-75597497	Hum_BBS	*SMAD6*	36.38	<0.0001	53.40	<0.0001	4.62	0.0321	−7.13 (3.32)	−0.10 (0.05)	−2.15	0.0321
AX-76351785	Hum_BBS	*WNT9B*	37.27	<0.0001	67.22	<0.0001	21.57	<0.0001	11.51 (2.48)	0.21 (0.04)	4.64	<0.0001
AX-75268181	Tib_BMD	*MCF2L*	4.30	0.0401	106.46	<0.0001	13.53	0.0003	−0.015 (0.004)	−0.15 (0.05)	−3.67	0.0003
AX-80813610	Tib_BMD	*CALCR*	4.24	0.0415	56.10	<0.0001	4.86	0.0298	0.008 (0.004)	0.10 (0.05)	2.21	0.028
AX-76099065	Tib_BMD	*SFRP4*	4.31	0.0400	65.23	<0.0001	8.55	0.0036	−0.016 (0.006)	−0.18 (0.06)	−2.92	0.0036
AX-77113061	Tib_BMD	*TMCO1*	4.45	0.0369	99.26	<0.0001	5.27	0.0221	0.008 (0.003)	0.11 (0.05)	2.30	0.0221
AX-75677174	Tib_BMD	*GPATCH1*	4.27	0.0406	61.13	<0.0001	10.84	0.0011	0.013 (0.004)	0.13(0.04)	3.29	0.0011
AX-77091655	Hum_BMD	*PODN*	20.70	<0.0001	51.56	<0.0001	11.53	0.0008	0.007 (0.002)	0.14 (0.04)	3.39	0.0008
AX-76351898	Hum_BMD	*WNT3*	19.82	<0.0001	77.58	<0.0001	13.81	0.0002	0.016 (0.004)	0.15 (0.04)	3.72	0.0002
AX-76351899	Hum_BMD	*WNT3*	19.82	<0.0001	77.58	<0.0001	13.81	0.0002	0.016 (0.004)	0.15 (0.04)	3.72	0.0002

^1^ BBS, bone breaking strength; BMD, bone mineral density; Tib, tibiotarsus; Hum, humerus; ^2^ Allele substitution effect per copy of the effect allele (minor allele); ^3^ SE, standard error; ^4^ Standardised regression coefficients expressed in SD unit.

**Table 3:** SNP effects analysis—Part 2: Genotypic values (least squares means) and additive and dominance effects obtained from the dominant-recessive model.

SNP	Trait ^1^	Candidate Gene	Genotypic Values			Homozygous Additive Allele Effect ^5^			Dominance Effect ^5^		
			AA ^2,3^ (SE ^4^)	AB ^2,3^ (SE)	BB ^2,3^ (SE)	Estimate (SE)	*t*-Value	*p*-Value	Estimate (SE)	*t*-Value	*p*-Value
AX-76044166	Tib_BBS	*MPP7*	155.33 (2.26) ^ab^	145.80 (5.85) ^b^	172.76 (7.25) ^a^	−8.71 (4.05)	−2.15	0.0320	−18.20 (5.45)	−3.35	0.0009
AX-76601713	Tib_BBS	*ACTR2*	162.77 (3.08) ^a^	156.79 (3.81) ^a^	143.10 (3.79) ^b^	9.83 (2.82)	3.49	0.0005	3.86 (4.03)	0.96	0.3392
AX-77276717	Tib_BBS	*TGFB2*	153.42 (2.25) ^a^	157.05 (3.06) ^a^	163.72 (5.10) ^a^	−5.15 (2.83)	−1.82	0.0694	−1.52 (3.73)	−0.41	0.6843
AX-76491534	Tib_BBS	*CCDC170*	159.13 (2.19) ^a^	144.04 (6.28) ^ab^	127.83 (8.09) ^b^	15.70 (4.42)	3.54	0.0004	0.56 (5.88)	0.096	0.9239
AX-76772658	Tib_BBS	*SOX6*	149.06 (2.65) ^b^	158.53 (2.58) ^a^	163.29 (3.90) ^a^	−7.11 (2.43)	−2.93	0.0035	2.36 (3.13)	0.75	0.4520
AX-75711229	Tib_BBS	*ASPN*	155.14 (1.94) ^b^	154.78 (5.29) ^b^	188.53 (13.11) ^a^	−16.70 (6.62)	−2.52	0.0120	−17.10 (8.02)	−2.13	0.0340
AX-75913642	Tib_BBS	*SOX9*	157.50 (1.93) ^a^	148.13 (4.83) ^ab^	124.13 (10.37) ^b^	16.70 (5.31)	3.14	0.0018	7.40 (6.44)	1.15	0.2506
AX-76772658	Hum_BBS	*SOX6*	127.04 (2.51) ^a^	116.24 (2.46) ^b^	119.38 (3.71) ^ab^	3.83 (2.31)	1.66	0.0984	−6.96 (3.02)	−2.31	0.0215
AX-77091655	Hum_BBS	*PODN*	118.01 (2.31) ^b^	120.73 (3.04) ^b^	132.21 (3.73) ^a^	−7.10 (2.31)	−3.07	0.0023	−4.38 (3.44)	−1.27	0.2043
AX-75597497	Hum_BBS	*SMAD6*	122.16 (2.08) ^a^	123.48 (3.64) ^a^	98.16 (6.97) ^b^	12.0 (3.71)	3.23	0.0013	13.30 (4.61)	2.88	0.0040
AX-76351785	Hum_BBS	*WNT9B*	115.73 (2.19) ^c^	124.86 (3.05) ^b^	139.61 (4.34) ^a^	−11.90 (2.54)	−4.70	<0.0001	−2.81 (3.49)	−0.80	0.4215
AX-75268181	Tib_BMD	*MCF2L*	0.263 (0.003) ^a^	0.253 (0.005) ^a^	0.228 (0.008) ^b^	0.017 (0.004)	3.92	0.0001	0.008 (0.006)	1.35	0.1768
AX-80813610	Tib_BMD	*CALCR*	0.256 (0.003) ^a^	0.258 (0.005) ^a^	0.273 (0.006) ^a^	−0.009 (0.004)	−2.24	0.0257	−0.007 (0.005)	−1.27	0.2051
AX-76099065	Tib_BMD	*SFRP4*	0.261 (0.003) ^ab^	0.265 (0.008) ^a^	0.235 (0.009) ^b^	0.013 (0.006)	2.32	0.0206	0.018 (0.006)	2.71	0.0071
AX-77113061	Tib_BMD	*TMCO1*	0.246 (0.005) ^a^	0.267 (0.004) ^a^	0.266 (0.004) ^a^	−0.01 (0.004)	−2.82	0.0050	0.011 (0.004)	2.51	0.0125
AX-75677174	Tib_BMD	*GPATCH1*	0.254 (0.003) ^b^	0.269 (0.005) ^a^	0.278 (0.007) ^a^	−0.012 (0.004)	−3.05	0.0024	0.004 (0.005)	0.56	0.5739
AX-77091655	Hum_BMD	*PODN*	0.164 (0.002) ^b^	0.167 (0.003) ^b^	0.178 (0.003) ^a^	−0.007 (0.002)	−3.53	0.0005	−0.004 (0.003)	−1.25	0.2117
AX-76351898	Hum_BMD	*WNT3*	0.166 (0.002) ^b^	0.176 (0.006) ^b^	0.206 (0.010) ^a^	−0.02 (0.005)	−3.84	0.0001	−0.009 (0.007)	−1.29	0.1991
AX-76351899	Hum_BMD	*WNT3*	0.166 (0.002) ^b^	0.176 (0.006) ^b^	0.206 (0.010) ^a^	−0.02 (0.005)	−3.84	0.0001	−0.009 (0.007)	−1.29	0.1991

^1^ BBS, bone breaking strength; BMD, bone mineral density; Tib, tibiotarsus; Hum, humerus; ^2^ AA or BB represents the homozygote of the other allele or effect allele, respectively. AB denotes the heterozygote (see Table 1 for the actual genotypes); ^3^ Means with different letters within a column differ significantly at *p* < 0.05; ^4^ SE, standard error; ^5^ Effect of the other allele (major allele).

## Data Availability

The data presented in this study are available on request from the corresponding author.

## Supplementary Materials

The following are available online at https://www.mdpi.com/article/10.3390/genes12050702/s1, Figure S1. Histograms of the residuals, Figure S2: Number of annotated SNPs per chromosome, Figure S3: Tree maps of significantly enriched Gene Ontology terms for genes associated with bone breaking strength, Figure S4: Tree maps of significantly enriched Gene Ontology terms for genes associated with bone mineral density, Table S1: Summary of the phenotypic measurements, Table S2: List of all extracted genes, Table S3: List of all enriched terms/pathways.
