# Supplementary material for: Identification and Functional Annotation of Genes Related to Bone Stability in Laying Hens Using Random Forests
Source: Genes (Basel). 2021 May 8;12(5):702. doi: 10.3390/genes12050702 (PMC8151682; doi:10.3390/genes12050702)
Supplement: Supplementary file 1 [file genes-12-00702-s001.zip › Supplement_proofed/Suppl_Figure_S1.pdf]

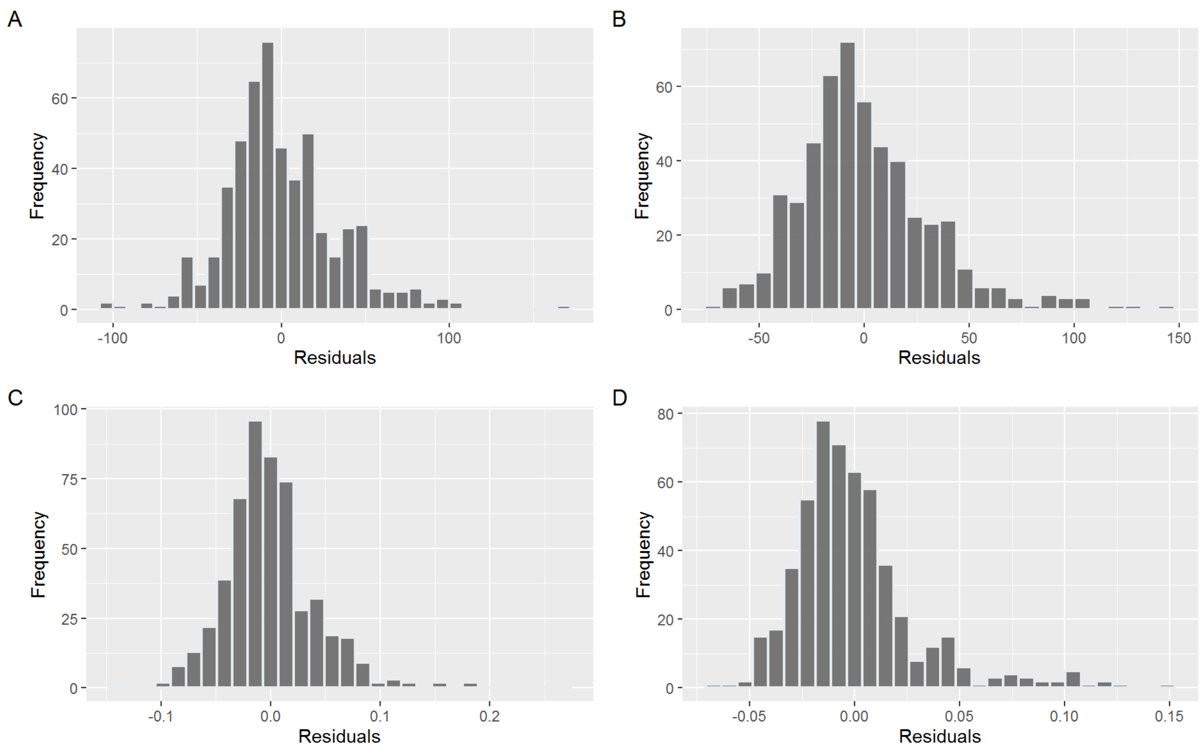

Figure S1: Histograms of the residuals for the bone breaking strengths of the tibiotarsus (**A**) and humerus (**B**), and the bone mineral densities of the tibiotarsus (**C**) and humerus (**D**).
