# Supplementary material for: Identification and Functional Annotation of Genes Related to Bone Stability in Laying Hens Using Random Forests
Source: Genes (Basel). 2021 May 8;12(5):702. doi: 10.3390/genes12050702 (PMC8151682; doi:10.3390/genes12050702)
Supplement: Supplementary file 1 [file genes-12-00702-s001.zip › Supplement_proofed/Suppl_Figure_S3.pdf]

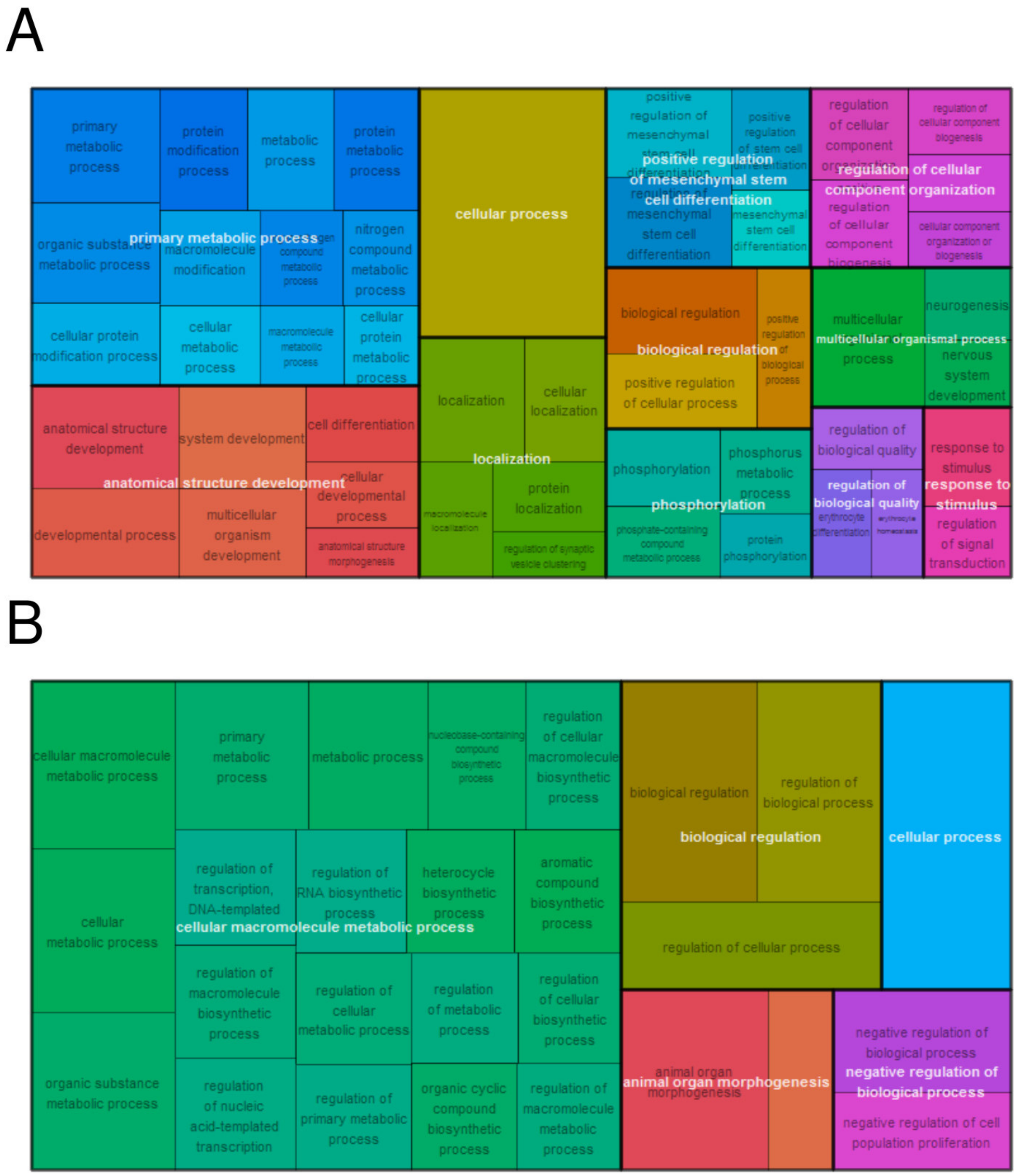

Figure S3: Tree maps of significantly enriched Gene Ontology (GO) terms of the category biological processes for genes associated with the bone breaking strengths of the tibiotarsus (**A**) and humerus (**B**). Each colour indicates a parent GO term drawn as a box in which the lower-level terms are plotted. The space filled by the terms is proportional to their -log$_{10}$ p-values.
