# Supplementary material for: Identification and Functional Annotation of Genes Related to Bone Stability in Laying Hens Using Random Forests
Source: Genes (Basel). 2021 May 8;12(5):702. doi: 10.3390/genes12050702 (PMC8151682; doi:10.3390/genes12050702)
Supplement: Supplementary file 1 [file genes-12-00702-s001.zip › Supplement_proofed/Suppl_Table_S1.pdf]

Table S1: Least squares means ± standard error for the bone breaking strengths and the bone mineral densities of the tibiotarsus and the humerus, taken verbatim from Jansen et al. (2020)[1].

	**Bone Breaking Strength (N)**		**Bone Mineral Density (g/cm²)**	
	Tibiotarsus	Humerus	Tibiotarsus	Humerus
WLA	137.34 ± 3.62	90.81 ± 3.43	0.211 ± 0.005	0.136 ± 0.003
R11	149.40 ± 3.54	109.94 ± 3.40	0.231 ± 0.005	0.156 ± 0.003
BLA	124.23 ± 3.58	138.64 ± 3.40	0.265 ± 0.005	0.197 ± 0.003
L68	211.57 ± 3.61	146.02 ± 3.45	0.327 ± 0.005	0.180 ± 0.003

[1] Reference: Jansen, S.; Baulain, U.; Habig, C.; Weigend, A.; Halle, I.; Scholz, A.M.; Simianer, H.; Sharifi, A.R.; Weigend, S. Relationship between Bone Stability and Egg Production in Genetically Divergent Chicken Layer Lines. *Animals* **2020**, 10, 850 (see Table 2).
